# Utilization of Electrical Impedance Spectroscopy and Image Classification for Non-Invasive Early Assessment of Meat Freshness

**DOI:** 10.3390/s21031001

**Published:** 2021-02-02

**Authors:** Sooin Huh, Hye-Jin Kim, Seungah Lee, Jinwoo Cho, Aera Jang, Joonsung Bae

**Affiliations:** 1 The Department of Electrical and Electronics Engineering, Kangwon National University, Chuncheon 24341, Korea; huhsi1024@kangwon.ac.kr (S.H.); hdmksm@kangwon.ac.kr (S.L.); 2 The Department of Applied Animal Science, College of Animal Life Science, Kangwon National University, Chuncheon 24341, Korea; mrk9929@kangwon.ac.kr (H.-J.K.); jyy003@kangwon.ac.kr (J.C.)

**Keywords:** freshness evaluation, electrical impedance spectroscopy (EIS), machine learning

## Abstract

This study presents a system for assessing the freshness of meat with electrical impedance spectroscopy (EIS) in the frequency range of 125 Hz to 128 kHz combined with an image classifier for non-destructive and low-cost applications. The freshness standard is established by measuring the aerobic plate count (APC), 2-thiobarbituric acid reactive substances (TBARS), and composition analysis (crude fat, crude protein, and moisture) values of the microbiological detection to represent the correlation between EIS and meat freshness. The EIS and images of meat are combined to predict the freshness with the Adaboost classification and gradient boosting regression algorithms. As a result, when the elapsed time of beef storage for 48 h is classified into three classes, the time prediction accuracy is up to 85% compared to prediction accuracy of 56.7% when only images are used without EIS information. Significantly, the relative standard deviation (RSD) of APC and TBARS value predictions with EIS and images datum achieves 0.890 and 0.678, respectively.

## 1. Introduction

Nowadays, customers pay more attention to the quality attributes of meat products, such as appearance, flavor, and nutrients, since there is wide variability in raw meat quality in commercial end products. Additionally, meat is highly susceptible to spoilage and contamination during the storage period, with freshness degradation caused by microbial spoilage and biochemical reactions. Therefore, there has been strong demand to assess the quality and freshness of meat to obtain reliable information about it and circumvent any possible food poisoning from spoilage [1,2,3].

Traditionally, human sensory evaluation was used to evaluate freshness by investigating the color, morphological features, and surfaces of meat. However, accuracy is vulnerable to the assessor’s bias and fatigue. Chemical substances detection and microbiological detection [4,5] using volatile basic nitrogen (VBN) [6,7], pH [8], 2-thiobarbituric acid reactive substances (TBARS) [9,10,11], and aerobic plate count (APC) [12] analysis methods have also been widely used to assess the freshness of meat due to their reliable, precise results. Nevertheless, these methods are destructive, time-consuming, complicated for experiments, and require skilled operators. Other alternatives to acquire early, low-cost, on-line, and non-destructive assessment, such as the electronic nose [13,14,15,16], image classification [17], torrymeter [18], and electrical impedance spectroscopy (EIS) technologies [19,20,21,22] have been actively studied. However, electric nose technology requires specific gas sensors and environmental conditions, and image classification suffers from low accuracy. Compared with torrymeter measurement, which indicates electrical impedance at a single frequency, EIS exploits multiple frequencies to characterize whether the biological object’s cell membranes are maintained to accurately determine the freshness of meat. In particular, EIS has shown a high correlation with meat aging [19,20], intramuscular fat (IMF) [21], and pH variation [22]. In addition, the moisture content was predicted by measuring the impedance of the meat [23].

Given that EIS and image classification technologies show outstanding advantages of using inexpensive measurements, and the accuracy of image classification can be compensated by combining it with another type of data set [17], in this paper, we propose an image classifier and EIS use method for non-invasive and accurate assessment of meat freshness. EIS (125 Hz—128 kHz) is measured according to the storage period (0, 8, 16, 24, 36, and 48 h) with meat images. The freshness standard is simultaneously established by measuring the APC and TBARS values to represent the correlation between EIS and meat freshness. We have primarily constituted the database with vector forms, including information of EIS and images adopted in the machine learning algorithms, which results in the non-invasive freshness assessment system with low cost and high precision.

## 2. Materials and Methods

### 2.1. Sample Preparation

**Figure 1:**
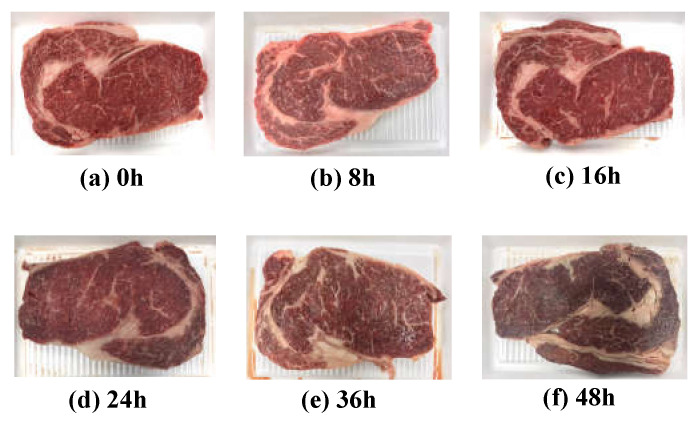
Images of beef loins over the elapsed storage period.

Quality grade 1+ and grade 1 beef loins and rounds were obtained from a local meat shop (Chuncheon, Korea). Each piece of beef was cut into 30 pieces (1 cm thick) using a sterile knife, placed on a Styrofoam tray and wrapped with low-density polyethylene film. A total of 120 samples was stored at 23 ± 2 °C to accelerate the degradation of freshness under a stress condition. As shown Figure 1, five pieces were randomly selected at each 0, 8, 16, 24, 36, and 48 h of storage. The EIS, APC values, TBARS values, proximate composition, and meat photos were acquired to analyze the correlation between freshness and EIS. Every measurement was performed five times with five different samples to average out any possible measurement errors.

### 2.2. EIS Measurement

**Figure 2:**
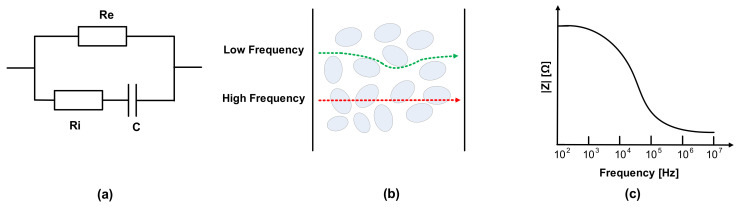
(**a**) Fricke model; (**b**) cell penetration at low and high frequencies; (**c**) impedance magnitude plot in frequency domain.

The electrical properties of meat can be evaluated by the mobility of ions in metabolism [24,25]. The dielectric constant is related to the material’s conductivity, and the current follows the cell in the extracellular fluid. Electrical impedance consists of the tissue components, intracellular fluids, and extracellular fluid of membranes. Na+ and Cl- ions exist in extracellular fluid, and the major anions of intracellular fluid are phosphate and proteins. Thus, extracellular and intracellular fluid can be regarded as electrolytes. Additionally, cell membranes can be modeled as capacitance; therefore, the impedance varies according to the frequency. The membrane’s capacitance and resistance of extracellular fluid and intracellular fluids are each considered as passive components C, Re, and Ri, as shown in Figure 2a [26,27,28,29,30]. As shown in Figure 2b, most currents cannot penetrate the cell membrane at a low frequency due to high resistance, whereas current at a high frequency flows through extracellular and intracellular spaces [31,32,33]. Therefore, impedance magnitude decreases as the frequency increases, as shown in Figure 2c, which means that in the case of impedance spectroscopy, impedance magnitudes according to the frequencies include the corresponding phase information of the cell membranes’ capacitance.

**Figure 3:**
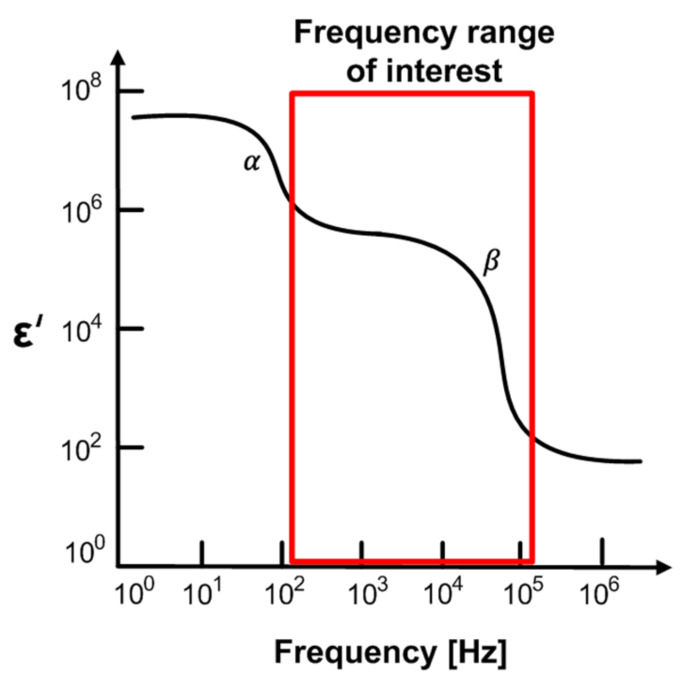
Dispersions of biological system.

Figure 3 shows dispersions of the biological system [34]. The α-dispersion is caused by polarization in the range of Hz to kHz, and β-dispersion in the range of kHz to MHz is mainly due to the Maxwell–Wagner effect, which is related to the interface polarization occurring in the system between two materials with different currents. In general, the α-dispersion and β-dispersion are more relevant to the cells states and are commonly used in impedance measurements for biological tissue studies. Especially, β-dispersion is related to the genetic properties of the cell membrane and the interaction between the cell membrane and the extracellular or intracellular electrolytes. This is directly associated with cell membranes’ behavior. It can be used in meat aging studies based on membrane integrity, because the oxidation of the phospholipid membrane layers and lysis occurring during aging makes the membrane porous and reduces the membrane’s insulating properties. As the meat ages, the impedance magnitude gradually tends to decrease, since the cell membranes are permeable. Therefore, we measure the freshness of meat through impedance measurements from 100 Hz to 1 MHz to discriminate the b-dispersion region.

**Figure 4:**
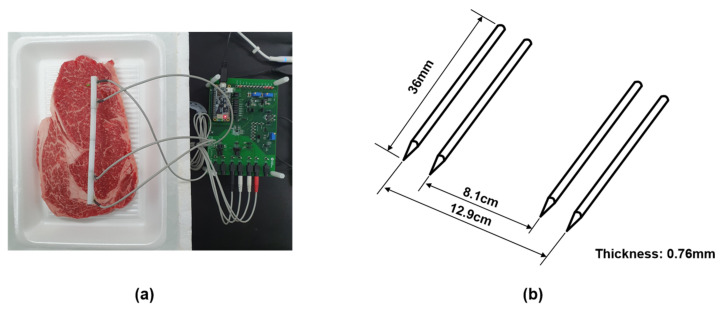
(**a**) The settings for the measurement. (**b**) Schematic diagrams of tetrapolar electrode.

A tetrapolar electrode interface was adopted to measure the EIS values to mitigate the effects of contact impedance between the electrode and meat [19]. A MAX30001 chipset was provided as a portable and low-cost impedance magnitude measurement method with electrode interface. It was controlled the frequency of 125–128 kHz at 1.1 V supply voltage (see datasheet MAX30001 [35]). We used needle electrodes made of stainless steel. The thickness of the electrode is 0.76 mm, and the length is 36 mm. The distance between the inner electrodes is fixed at 8.1 and 12.9 cm for the outer electrodes. The electrodes were inserted to a depth of 0.5 cm on the meat, as shown in Figure 4. Impedance is obtained for each measurement at seven frequencies of 125 Hz, 250 Hz, 500 Hz, 2 kHz, 8 kHz, 80 kHz, and 128 kHz at 8 μA injection AC current magnitude.

As the previous study results [36,37] measured the impedance spectroscopy (40 Hz to 110 MHz) on beef with storage time from one day to 14 days, there were no significant differences in impedance value at the frequency more than 100 kHz. Furthermore, the impedance value is almost constant at a frequency of more than 2 MHz (outside of the β-dispersion region). Since the target application is for a portable, rapid, and straightforward assessment with battery-powered instrumentation, we determined that measuring up to a frequency around 100 kHz would be enough to obtain the assessment information. In addition, we obtained the magnitude information from the real and imaginary parts of the measured impedance for the compactness of the portable system.

### 2.3. APC Measurement

First, 90 mL of saline was added to 10 g of the sample and homogenized for 40 s using a stomacher (Bag Mixer 400, Interscience, France). The homogenate was diluted by each dilution factor using saline. One mL of homogenate was dispensed onto 3 M Petrifilm (aerobic count plate, 3 M, Maplewood, MN, USA) according to the manufacturer’s method and cultured at 37 °C for 48 h to count colonies.

### 2.4. TBARS Measurement

Then, 50 μL 7.2% BHA was added to 5 g of sample, 15 mL of distilled water was added, and a homogenizer was used for homogenization [38], and 2 mL of a 20 mM TBA (15% TCA dissolution) reagent was added to 1 mL of the homogenate. After mixing, the mixture was heated at 90° C for 15 min. After heating, it was cooled in cold water and centrifuged for 10 min at a rate of 2000× *g*. After that, the supernatant was measured at 531 nm using a UV/VIS spectrophotometer (Molecular Device, M2e, Sunnyvale, CA, USA). A blank sample was measured in the same manner by adding distilled water instead of the sample. The following calculation formula (1) was used for the TBARS value in the sample.

TBARS(mg malondialdehyde/kg) = (absorbance of the sample − absorbance of the blank) × 5.88(1)

### 2.5. Proximate Composition Measurement

The moisture content was determined by oven drying at 105 °C, and the crude protein content was determined by the Kjeldahl method. The crude fat content was assessed by solvent extraction, and the crude ash was analyzed by burning the samples in a furnace at 550 °C [39].

### 2.6. Statistical Analysis

One-way ANOVA was performed on all the experiment results using the SAS v9.4 (SAS Institute Inc., Cary, NC, USA). The significance of the mean values was verified at the 5% level by the Tukey method. The correlation between impedance and freshness over the storage period was analyzed by Pearson’s correlation coefficient (r) using an SAS program. The SPSS program (IBM, Armonk, NY, USA) was used for the principal component analysis (PCA) of the composition and impedance measurement method.

## 3. Results and Discussion

### 3.1. EIS Results

**Figure 5:**
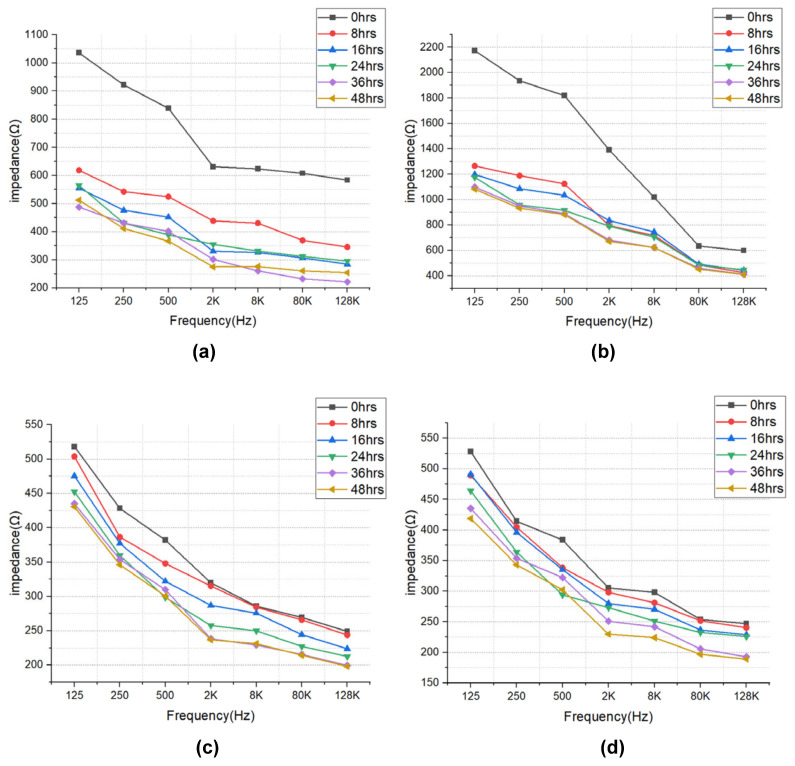
EIS of (**a**) beef loin (quality grade 1+), (**b**) beef loin (quality grade 1), (**c**) beef round (quality grade 1+), and (**d**) beef round (quality grade 1) measured over the storage periods.

**Figure 6:**
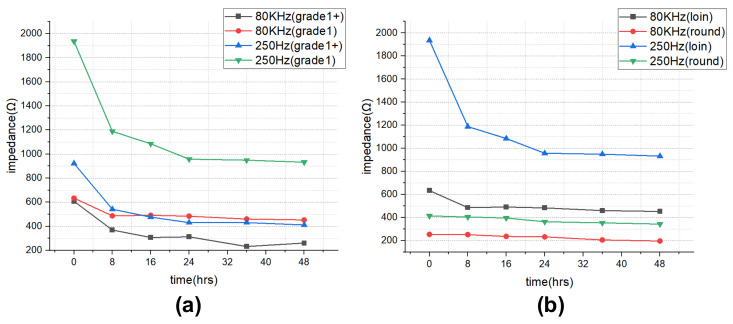
Impedance over the storage periods showing differences between (**a**) quality grade 1+ and 1, and (**b**) loin and round.

The impedance results of beef loin (grade 1+, grade 1) and round (grade 1+, grade 1) are shown in Figure 5. The impedance value tends to decrease with increasing storage period. This is because the insulating cell membranes become permeable by membrane destruction as the storage time increases, which lowers the impedance. In addition, as the frequency increases, the movement of ions in the extracellular tends to gradually penetrate the intracellular along insulating membranes, which makes the impedance decrease. Figure 6 compares the measured results by type of quality grade, and loin/round cut at frequencies of 80 kHz and 500 Hz. Figure 6a shows differences between quality grades. Overall, the impedance of quality grade 1 was higher than grade 1+. However, it is difficult to correlate the grade with impedance measurements, because the grade of beef is determined by combinations of various factors, such as marbling, meat color, fat color, texture, and maturity (Ministry of Agriculture, Food and Rural Affairs, 2018). It is challenging to infer all these values by impedance measurement. Figure 6b shows the differences between meat cuts (loin and round). The impedance is significantly higher in loin than round, since loin contains more fat components than round, given that fat component has less moisture and has larger impedance [40].

### 3.2. APC and TBARS Results

**Figure 7:**
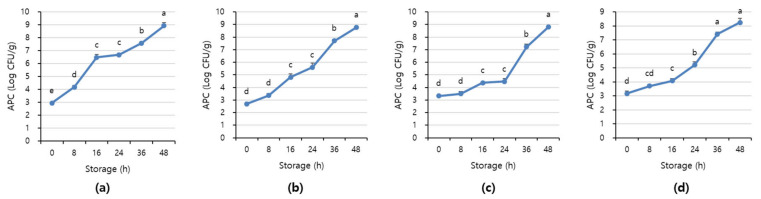
The APC level of (**a**) beef loin (quality grade 1+), (**b**) beef loin (quality grade 1), (**c**) beef round (quality grade 1+), and (**d**) beef round (quality grade 1) over the storage periods at room temperature (23 °C). ^a–e^ Different superscript letters above bars indicate a significant difference at *p* < 0.05.

**Figure 8:**
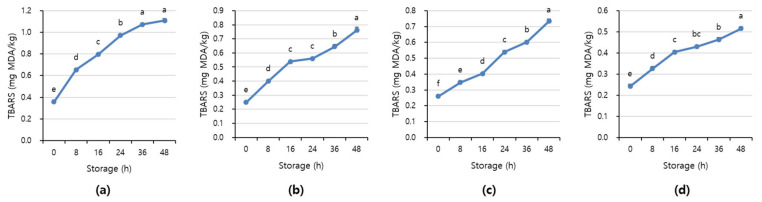
The TBARS value of (**a**) beef loin (quality grade 1+), (**b**) beef loin (quality grade 1), (**c**) beef round (quality grade 1+), and (**d**) beef round (quality grade 1) over the storage periods at room temperature (23 °C). ^a–f^ Different superscript letters above bars indicate a significant difference at *p* < 0.05.

Figure 7 and Figure 8 shows APC and TBARS values over the storage period. Initially, the APC in the beef was 2.70–3.16 Log CFU/g, determined to be fresh. However, as the storage period increased, the APC and TBARS increased in all treatments (*p* < 0.05). When APC in meat is in the range of 6–8 Log CFU/g, the meat starts to decay, and an off odor and viscous substances increase [41]. In addition, beef having 6.7 Log CFU/g (5 × 10^6^ CFU/g) or less of the APC should be distributed to meat shop following the Recommended Criteria for Microbial Testing in Meat (Ministry of Food and Drug Safety, 2018) in Korea [42]. Starting from 36 h of storage, all treatment exceeded the recommended APC level and showed more than 7 Log CFU/g, which is considered to be spoiled. The TBARS values in all treatment were 0.46–1.07 mg MDA/kg, and the 1+ grade loin with high fat content showed the highest TBARS value.

The TBARS value, which indicates the degree of rancidity of meat maintenance, is a measure of the intensity of the red color produced by the reaction of malondialdehyde (MDA) and thiobarbituric acid (TBA) generated by the oxidation of fat [43]. The range of TBARS in which the rancid off-odor that occurs in beef can be sensed varies from 0.6 to 2.0 mg MDA/kg [44]. The maximum allowable rancid off-odor in beef was 2 mg MDA/kg, and a value of more than 1.2 mg MDA/kg means it is completely spoiled [45,46].

### 3.3. Correlation Between EIS and Microbiological Detection

Table 1 shows correlations between EIS and microbiological detection values. There was a negative correlation between impedance and the APC and TBARS values. However, in the case of beef round, there was a significant correlation. The interface (−0.629 ≤ r ≤ −0.850, *p* < 0.01) showed a higher correlation for 1+ grade beef loin. In addition, as the frequency increases, the correlation increases, and the TBARS value is highly correlated at 128 kHz (r = −0.850, *p* < 0.01) and 80 kHz (r = −0.852, *p* < 0.01). There is a more significant correlation at frequencies above kHz, which corresponds to the β-dispersion region directly related to meat aging. On the other hand, the correlation between APC and TBARS tends to increase as the frequency of grade 1 loin decreases, and in particular, the TBARS showed the highest correlation (r = −0.610, *p* < 0.01) with 500 Hz. In the 1+ grade beef round, the 128 K frequency showed the highest correlation (r = −0.445, *p* < 0.05), and the grade 1 beef round showed the highest correlation with the 128 K frequency with APC (r = −0.736, *p* < 0.01).

### 3.4. Proximate Composition Results

Table 2 shows the results of the proximate composition of beef loin (grade 1+, grade 1) and round (grade 1+, grade 1) used in the experiment. The grade 1+ and 1 were carcass quality grade in Korea, which means marbling score, firmness, lean meat color, fat color, and maturity (1++; the highest grade, 1, 2, and 3; the lowest grade) [47]. The moisture and crude protein were significantly higher in the round region than in the loin region. On the other hand, in crude fat, the loin portion was higher than the round portion, and in particular, the grade 1+ loin showed the highest crude fat content (*p* < 0.05). There was no significant difference in the period of review by part and by grade. These results were similar to the previously reported results [42,48,49,50,51].

### 3.5. Correlation between EIS and Composition Analysis

Table 3 shows correlations between the composition of beef and the impedance values. The impedance value and the moisture and crude protein content in the beef show a negative correlation, and the crude fat content shows a positive correlation. Crude ash is not associated with impedance. Impedance shows a high correlation with crude fat and crude protein. The frequency of 128 kHz shows the highest correlation with crude fat, and frequencies from 125 to 80 kHz show the highest correlation with crude protein.

**Figure 9:**
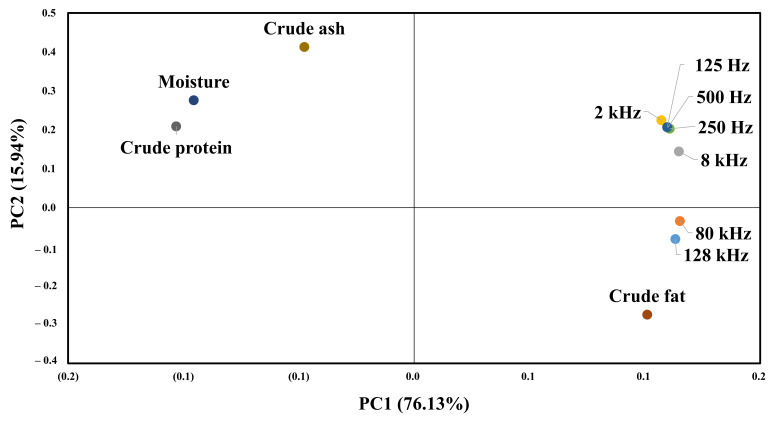
PCA for the proximate composition results and EIS.

Figure 9 shows the results of principal component analysis for the composition of beef and impedance spectroscopy. The impedance value for each frequency and the value of the first principal component for the general component are 76.13%. The value of the second principal component is 15.94%, which means that the first principal component can explain 76.13% of the data. All impedance values and crude fat content are distributed on the right side of the first main component, so that the higher the crude fat, the higher the impedance value. The second main component indicates the difference by frequency, and frequencies below 8 K show a high correlation with crude protein and moisture. In contrast, the 128 and 80 K frequencies show a high correlation with crude fat.

## 4. Image Classification with EIS Results

As a result of the experiment, it was confirmed that APC, TBARS, and composition measured by the conventional method were statistically correlated with EIS, which means that impedance measurements can predict the freshness and composition of meat in daily life. Using image data with EIS, machine learning was performed using loin (grade 1+) data, which had the highest correlation.

**Figure 10:**
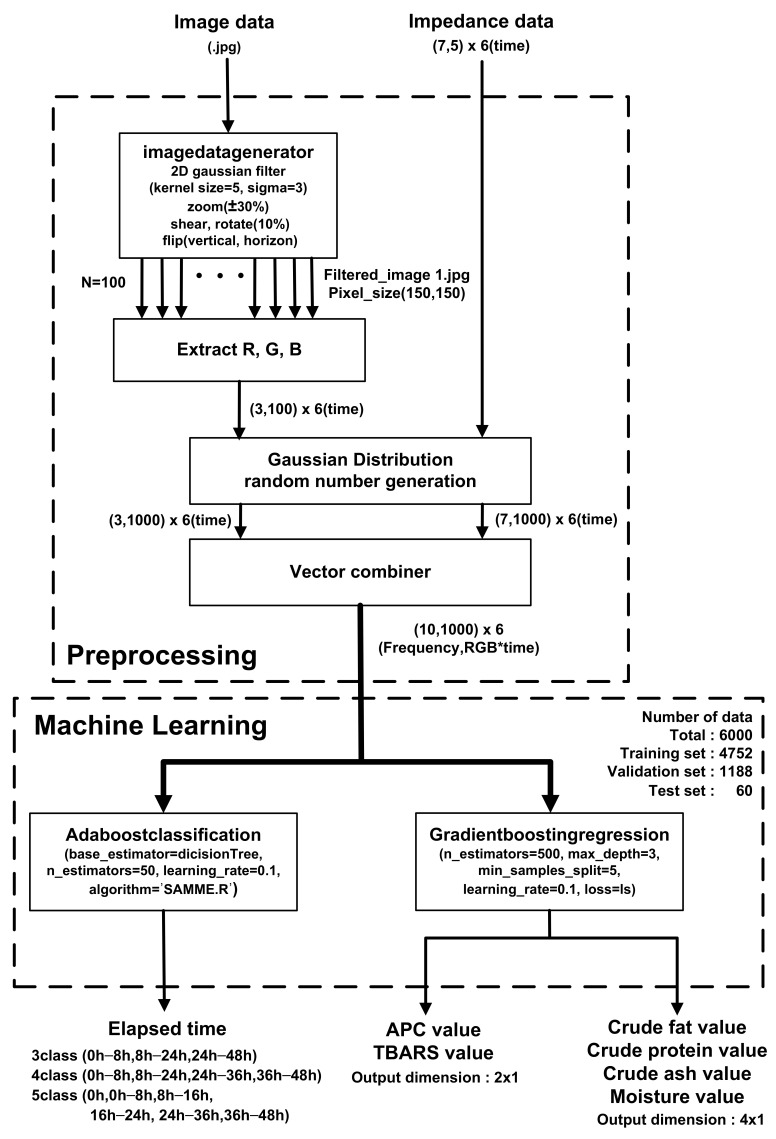
Utilization of EIS and image classification with preprocessing and AdaBoost classification and gradient boosting regression machine learning algorithms.

Figure 10 shows a block diagram of the prediction algorithm. In pre-processing, filtered images are generated for each storage period (0, 8, 16, 24, 36, and 48 h) for a sufficient amount of training data. After applying a 2D Gaussian filter with kernel size = 5 and sigma = 3, ±30% zoom, 10% shear, 10% rotation, vertical flip, and horizontal flip are applied, and 100 images with a size of (150,150) pixels are used to extract the R, G, and B of each pixel with (3100*6) vectors. Then, using Gaussian distribution random number generation and a vector combiner, the averaged R, G, and B values of the images and EIS were combined to generate (10,1000) vectors over the storage periods. The 79.2%, 19.8%, and 1% of the generated data set were used for training, validation, and testing, respectively. Prediction was performed with an AdaBoost (adaptive boosting) classifier and gradient boosting regressor based on a decision tree learner. In the boosting method, a gradient boosting method is performed in which the error of the previous tree is determined through a negative gradient while creating trees sequentially and an Adaboost method is performed in which the classifier adaptively changes the wrong part and places a high weight on the classifier with many misclassified data and reflects it in the next sample classification [52]. The classifier estimates the storage periods of meat at room temperature based on combined vectors from EIS and meat images. The estimation accuracy was evaluated according to the number of classes (3, 4, and 5) that can distinguish between the complete corruption of APC and the pretense of TBARS. The parameters max_depth = 1, n_estimator = 50, learning_rate = 0.65, and Algorithm = SAMME.R were used for fast converges with low errors with fewer boosting iterations. When predicting a storage period with three classes (fresh (0–8 h)/rancid (8–24 h)/spoiled (24–48 h), the use of EIS significantly improves accuracy by up to 85%. In contrast, the accuracy is 56.7% when only images are used in the classifier, as shown in Table 4.

Second, APC and TBARS values were estimated with a gradient boosting regression algorithm. The parameter setting was n_estimators = 600, representing the number of boosting steps to be performed, max_depth = 3, which is the number of nodes in the tree, min_samples_split = 5, which is the minimum number of samples required to split an internal node, and learning_rate = 0.1 with least squares regression. The estimated value was evaluated with a relative standard deviation (RSD). N is the number of samples in the test set, $y_{i}$ is the actual value, and $\hat{y_{ı}}$ is the predicted value in Equation (2). It was used to compare data sets with different units of measurement. $\bar{y}$ in Equation (3) is the average of the actual values.

(2) $$\mathrm{RMSE}=\sqrt{\frac{1}{N}\overset{N}{\underset{i=1}{\sum }}{\left(y_{i}-\hat{y_{ı}}\right)}^{2}}$$

(3) $$\mathrm{RSD}\;=\;\frac{\mathrm{RMSE}}{\bar{y}}\times 100$$

Table 5 shows that the RSD value is much lower when vectors are combined with EIS and image information than when only EIS and pictures are applied. The RSD value of APC is 1.197, and value of TBARS is 0.678 at Image with EIS.

Furthermore, given that moisture, crude fat, and crude protein are positively correlated with the EIS in Section 3, the gradient boosting regression algorithm is also used to estimate the component analysis. As shown in Table 6, the RSD value of moisture, crude fat, and crude protein shows less than 5%, while the RSD value of crude ash is 11.6%.

Although the comparisons with other works is not easy since the types of the meat, what to predict, number of the samples, and classes affect the accuracy of the prediction, the proposed scheme of combination of image classification and EIS is comparable with the state-of-the-art works using image classification [17,53], and moisture content prediction using EIS [23].

## 5. Conclusions

EIS has high usability, rapidity, and non-destructiveness compared to conventional meat freshness assessment methods such as sensory evaluation, microbiological detection, and chemical substances detection. Impedance spectroscopy in the 2–128 kHz range over the storage period shows a significant correlation with APC and TBARS values, which are reliable indicators of corruption. In addition, crude fat, crude protein, and moisture of beef also have a positive correlation. These results show the suitability of impedance information to assess the freshness of beef. Based on these results, it was possible to predict the storage period or numeric values of APC/TBARS, and composition analysis through a machine learning algorithm using combined vector information from images of beef and EIS and showed 10–20% higher accuracy than the case of prediction with only image information. In addition, it showed meaningful result of RSD. As a further work, higher accuracy can be expected when collecting data on both magnitude and phase when measuring EIS and expanding the frequency range up to a few MHz with the sophisticated algorithms such as convolution neural network (CNN) and recursive neural network (RNN).

## Figures and Tables

**Table 1:** Correlation coefficients between each impedance at different frequencies and freshness of beef.

Meat	Freshness	Frequency (Hz)						
		128 K	80 K	8 K	2 K	500	250	125
**Beef Loin** **(Quality Grade 1+)**	**APC**	−0.784 **	−0.788 **	−0.796 **	−0.778 **	−0.675 **	−0.659 **	−0.629 **
	**TBARS**	−0.850 **	−0.852 **	−0.837 **	−0.804 **	−0.726 **	−0.715 **	−0.669 **
**Beef Loin** **(Quality Grade 1)**	**APC**	−0.350	−0.327	−0.383 *	−0.409 *	−0.483 **	−0.485 **	−0.466 **
	**TBARS**	−0.481 **	−0.425 *	−0.490 **	−0.509 **	−0.610 **	−0.617 **	−0.605 **
**Beef Round** **(Quality Grade 1+)**	**APC**	−0.408 *	−0.416 *	−0.387 *	−0.412 *	−0.066	−0.022	−0.001
	**TBARS**	−0.445 *	−0.468 **	−0.412 *	−0.464 **	−0.146	−0.073	−0.055
**Beef Round** **(Quality Grade 1)**	**APC**	−0.736 **	−0.702 **	−0.522 **	−0.476 **	−0.303	−0.376 *	−0.422 *
	**TBARS**	−0.648 **	−0.628 **	−0.539 **	−0.454 *	−0.343	−0.334	−0.422 *

* *p* < 0.05, ** *p* < 0.01.

**Table 2:** Proximate composition of beef loin and round.

Proximate Composition (%)	Beef Loin		Beef Round		SEM
	Grade 1+	Grade 1	Grade 1+	Grade 1	
**Moisture**	62.62 ^c^	64.22 ^b^	70.72 ^a^	70.51 ^a^	0.352
**Crude Fat**	17.59 ^a^	15.02 ^b^	6.43 ^c^	6.04 ^c^	0.313
**Crude Protein**	18.87 ^b^	19.67 ^b^	21.66 ^a^	22.28 ^a^	0.314
**Crude Ash**	0.91 ^a^	1.08 ^a^	1.19 ^a^	1.18 ^a^	0.078

^a–^^c^ Means within a row with a different superscript differ significantly at *p* < 0.05.

**Table 3:** Correlation coefficients between each impedance at different frequencies and composition analysis results.

Proximate Composition	Frequency (Hz)						
	128 K	80 K	8 K	2 K	500	250	125
**Moisture**	−0.826 **	−783 **	−0.635 **	−0.483 *	−0.562 **	−0.571 **	−0.556 *
**Crude Fat**	0.861 **	0.832 **	0.680 **	0.539 *	0.604 **	0.613 **	0.601 **
**Crude Protein**	−0.845 **	−0.857 **	−0.724 **	−0.626 **	−0.652 **	−0.665 **	−0.658 **
**Crude Ash**	−0.432	−0.387	−0.227	−0.155	−0.147	−0.146	−0.145

* *p* < 0.05, ** *p* < 0.01.

**Table 4:** Comparison of storage period prediction accuracy between image input only and image input with EIS information.

	5 Class	4 Class	3 Class
**Accuracy (%)**			
**Image**	20	43.66	56.66
**Image + EIS**	55.0	61.66	85.0

**Table 5:** Comparison of APC/TBARS value prediction RSD among image input only, EIS information only, and image input with EIS information.

		Image	EIS	Image + EIS
**RSD**	**APC**	2.012	1.555	1.197
	**TBARS**	1.802	0.890	0.678

**Table 6:** Composition value prediction and RSD with EIS information.

	Crude Fat	Crude Protein	Crude Ash	Moisture
**RSD**	4.571	2.409	11.574	0.872

## Data Availability

Data available on request from the authors. The data that support the findings of this study are available from the corresponding author, Joonsung Bae, upon reasonable request.
